# Does the revised cardiac risk index predict cardiac complications following elective lung resection?

**DOI:** 10.1186/1749-8090-8-220

**Published:** 2013-12-01

**Authors:** Robin Wotton, Andrea Marshall, Amy Kerr, Ehab Bishay, Maninder Kalkat, Pala Rajesh, Richard Steyn, Babu Naidu

**Affiliations:** 1 Department of Thoracic Surgery, Heart of England NHS Foundation Trust, Bordesley Green East, Birmingham B9 5SS, UK; 2 Warwick Medical School Clinical Trials Unit, Warwick Medical School, University of Warwick, Coventry CV4 7AL, UK; 3 University of Birmingham, Office 8 First Floor Research Laboratories, Mindelsohn Way, Edgbaston, Birmingham B15 2WB, UK

**Keywords:** Risk factors, Lung, Surgery, Complications, Cardiac arrhythmia

## Abstract

**Background:**

Revised Cardiac Risk Index (RCRI) score and Thoracic Revised Cardiac Risk Index (ThRCRI) score were developed to predict the risks of postoperative major cardiac complications in generic surgical population and thoracic surgery respectively. This study aims to determine the accuracy of these scores in predicting the risk of developing cardiac complications including atrial arrhythmias after lung resection surgery in adults.

**Methods:**

We studied 703 patients undergoing lung resection surgery in a tertiary thoracic surgery centre. Observed outcome measures of postoperative cardiac morbidity and mortality were compared against those predicted by risk.

**Results:**

Postoperative major cardiac complications and supraventricular arrhythmias occurred in 4.8% of patients. Both index scores had poor discriminative ability for predicting postoperative cardiac complications with an area under receiver operating characteristic (ROC) curve of 0.59 (95% CI 0.51-0.67) for the RCRI score and 0.57 (95% CI 0.49-0.66) for the ThRCRI score.

**Conclusions:**

In our cohort, RCRI and ThRCRI scores failed to accurately predict the risk of cardiac complications in patients undergoing elective resection of lung cancer. The British Thoracic Society (BTS) recommendation to seek a cardiology referral for all asymptomatic pre-operative lung resection patients with > 3 RCRI risk factors is thus unlikely to be of clinical benefit.

## Background

Risk models aid the decision- making process for patients contemplating surgery. Surgeons are able to provide accurate information on a more individual basis facilitating the consent process prior to surgery. Several risk models and stratification tools have been developed identifying risk factors for cardiac complications after adult general thoracic surgery. The Revised Cardiac Risk Index score (RCRI) was devised from a broad surgical population and included only a small group of patients who underwent thoracic surgery (12%) [1]. Brunelli and colleagues recalibrated the RCRI specifically for lung resection surgery generating the Thoracic Revised Cardiac Risk Index score (ThRCRI) [2]. The new United Kingdom (UK) and European guidelines for the radical management of patients with lung cancer have incorporated the RCRI as a risk assessment tool for the selection for surgery for patients who are not perceived to have active cardiovascular disease [3,4]. The discriminative and predictive ability of RCRI in lung resection surgery alone has not been reported in the UK population.

The recent British Thoracic Society (BTS) guidelines, place increasing emphasis on shared decision making, with individual risk scores being discussed with every patient. The risk of life threatening cardiac complications is low but the development of supraventricular arrhythmias is common. Both the RCRI and the ThRCRI were developed to predict the risk of development of supraventricular arrhythmias. However, these ‘minor’ complications are similarly associated with a prolonged stay in hospital and slower recovery to more significant adverse events. They may be as important when discussing operative risk with patients, as the small risk of death from more life threatening complications.

No validated system exists to provide patients with the risk of these more common complications. In this study we aim to test whether the RCRI and its modification the ThRCRI can accurately predict all cardiac complications after elective lung resection surgery [5,6].

## Methods

In this prospective observational study, consecutive patients who underwent thoracotomy and lung resection between October 2007 and October 2010 at a large UK regional thoracic centre were observed until discharge for a minimum of 30 days post admission.

Lung resection was only carried out on those patients with stable cardiovascular disease. ACC/AHA guidelines were instituted for any patient with an unstable cardiac condition or prohibitive cardiac risk and surgical resection reconsidered.

All patients were managed daily by a specialised thoracic team comprising of a senior thoracic surgeon and junior doctors on a dedicated Thoracic surgical High Dependency Unit (HDU) and Thoracic wards. They received daily physiotherapy programme from the first postoperative day which included sitting out of bed, early mobilisation, progressive exercise, deep breathing exercises, assisted coughing and nebulisation and humidification.

Data collected prospectively included demographic information, diagnosis, surgery type, smoking and alcohol intake, American Society of Anaesthesiologists (ASA) score, performance status, breathlessness score, pre-operative self reported exercise capacity, Chronic Obstructive Pulmonary Disease (COPD) status, co morbidities, pre-operative blood creatinine levels, pre-operative Forced Expiratory Volume in one second (FEV_1_), postoperative predicted (ppo) FEV_1_, length of in-hospital and HDU stay, intensive care unit (ITU) admission. All patients were screened in a pre-operative assessment clinic to identify any previous history of cardiac disease or arrhythmia, as part of routine work up before surgery. This included taking a detailed cardiac history as part of full evaluation of the patient in preparation for surgery. Specific questions include any history of myocardial infarction, angina, hypertension, diabetes mellitus, previous cardiac surgery or percutaneous intervention, permanent pacemaker or stroke.

Post-operative major cardiac complications included pulmonary oedema [diagnosed by chest radiograph consistent with appropriate clinical setting], myocardial infarction (MI) [diagnosed by rise in serum troponin and changes on electrocardiogram], ventricular fibrillation (VF) arrest, supraventricular arrhythmia, atrial fibrillation (AF) and mortality occurring within 30 days. All major lung resections (segmentectomy, lobectomy, bi-lobectomy and pneumonectomy) and a proportion of wedge resections spent at least 24 hours in a thoracic surgery high-dependency unit. Standard bedside clinical monitoring included a cardiac monitor with continuous electrocardiography. Cardiac arrhythmias are thus detected immediately. The remaining patients nursed on the ward had frequent regular bedside observations taken to identify any new arrhythmias.

The RCRI and ThRCRI were calculated retrospectively for all participants. The RCRI was calculated as originally described, with 1 point given for each of the 6 independent predictors: high-risk type of surgery, history of ischaemic heart disease (IHD), history of congestive heart failure (CCF), history of cerebrovascular disease (CVA), preoperative treatment with insulin, and preoperative serum creatinine of ≥2.0 mg/dL [1]. The patients were then grouped into those with 0, 1, 2, or ≥3 of these predictors. As Lee described all thoracic procedures as “high risk” our data set are noted to have a score of at least 1 as a result [1].

The ThRCRI was calculated as originally described [2]. The score comprises four predictors with associated weighting: ischaemic heart disease (scoring 1.5 points); history of cerebrovascular disease (1.5 points); serum creatinine level greater than 2 mg/dL (1 point) and pneumonectomy (1.5 points). The patients are grouped into four classes.

### Statistical methods

Results are expressed in percentage for categorical variables, and median, interquartile range and full range for non-normally distributed continuous variables.

Univariate analyses using Pearson’s chi-square test or Fisher’s exact test, where appropriate, for categorical factors and a Wilcoxon rank sum test for continuous factors, were performed to assess their relationship with Cardiac complications.

The performance of the cardiac risk scores (RCRI and ThRCRI) in predicting post-operative cardiac complications within 30 days were assessed using the area under a receiver operator characteristic (ROC) curve and associated 95% confidence intervals [7] for assessing model discrimination and the R^2^ measure of goodness of fit as a measure of predictive ability from a logistic regression model with the score included as a categorical variable. All analyses were performed using the SAS statistical package version 9.2 (SAS Institute, Inc, Cary NC).

## Results

Data from a total of 703 patients who underwent lung resection surgery within a single institution were analysed. The characteristics of these patients are displayed in Table 1. Ninety-five patients (14%) had a history of ischemic heart disease or congestive heart failure. The median age of the patients was 68 years (range 14-89) and 401 patients (57%) were male. The procedures performed were 385 (55%) lobectomies, 158 (22%) wedge excisions, 67 (10%) pneumonectomies, 42 (6%) segmentectomies, 31 (4%) explorations and 20 (3%) sleeve resections. Risk scores were calculated for all patients. An RCRI score = 3 was recorded in 6.8% (68 patients) and a score = 4 in 19 (2.7%) of study population. The ThRCRI scores were also calculated. A ThRCRI score = 2.5 (24 patients, 3.4%); score = 3 (10 patients, 1.4%); score = 4 (2 patients, 0.3%).

**Table 1:** Frequency of all post-operative cardiac complications by patient characteristics

**Variable**	**Value**	**Total**	**Post operative complications**	
			**Yes**	**No**
		**n (%)**	**n (%)**	**n (%)**
**Total**		703	34 (5%)	669 (95%)
**Age (in years) (p = 0.06)**	< 55	126 (18%)	4 (3%)	122 (97%)
	55-65	177 (25%)	4 (2%)	175 (98%)
	> 65	400 (57%)	26 (7%)	374 (93%)
**Gender (p = 0.20)**	Male	401 (57%)	11 (4%)	291 (96%)
	Female	401 (43%)	23 (6%)	378 (94%)
**BMI (p = 0.41)**	Median	26.0	26.4	26.0
	(Interquartile range)	(23.4-29.0)	(23.7-31.5)	(23.4-29.0)
**American Society of Anesthesiologists score (p = 0.14)**	1 or 2	293 (42%)	10 (3%)	283 (97%)
	3 or 4	410 (58%)	24 (6%)	386 (94%)
**Performance status (p = 0.16)**	2	659 (94%)	30 (5%)	629 (95%)
	3	44 (6%)	4 (9%)	40 (91%)
**Dyspnoea score (p = 0.14)**	2	600 (85%)	26 (4%)	574 (96%)
	3	103 (15%)	8 (8%)	95 (92%)
**Operation (p = 0.76)**	Other	636 (90%)	32 (5%)	604 (95%)
	Pneumonectomy	67 (10%)	2 (3%)	65 (97%)
**Lung Cancer (p = 0.35)**	No	63 (9%)	1 (2%)	62 (98%)
	Yes	640 (91%)	33 (5%)	607 (95%)
**Comorbidity (p = 0.62)**	None	18 (3%)	0	18 (100%)
	1-2	352 (50%)	17 (5%)	335 (95%)
	3+	333 (47%)	17 (5%)	316 (95%)
**Creatinine group (p = 0.54)**	Low	639 (91%)	30 (5%)	609 (95%)
	High	64 (9%)	4 (6%)	60 (94%)
**Diabetes (p = 0.60)**	No	616 (88%)	29 (5%)	587 (95%)
	Yes	87 (12%)	5 (6%)	82 (94%)
**Cholesterol (p > 0.99)**	No	686 (98%)	34 (5%)	652 (95%)
	Yes	17 (2%)	0	17 (100%)
**Hypertension (p = 0.69)**	No	453 (64%)	23 (5%)	430 (95%)
	Yes	250 (36%)	11 (4%)	239 (96%)
**Peripheral vascular disease (p = 0.50)**	No	689 (98%)	33 (5%)	656 (95%)
	Yes	14 (2%)	1 (7%)	13 (93%)
**Cerebrovascular accident (p = 0.05)**	No	685 (91%)	31 (5%)	654 (95%)
	Yes	18 (9%)	3 (17%)	15 (83%)
**Alcohol (p > 0.99)**	No	702 (99%)	34 (5%)	668 (95%)
	Yes	1 (1%)	0	1 (100%)
**Smoking (p = 0.41)**	No	420 (60%)	18 (4%)	402 (96%)
	Yes	283 (40%)	16 (6%)	267 (94%)
**Chronic obstructive pulmonary disease (p = 0.67)**	No	538 (77%)	25 (5%)	513 (95%)
	Yes	165 (23%)	9 (5%)	156 (95%)

In our series, there were 5 (1%) major post-operative cardiac complications. Two patients developed pulmonary oedema, two had an acute myocardial infarction and one had a ventricular fibrillation arrest.

Two of these 5 (40%) patients died due to their cardiac complications in hospital. In a further 14 patients, who died from pulmonary complications, none developed a concomitant cardiac complication.

There were a further 29 (4%) patients who developed atrial fibrillation or supraventricular tachycardia after surgery. Patients who developed a post-operative cardiac complication had a significantly longer in hospital length of stay (median 9 days, interquartile range 7-13 days) compared to those without cardiac complications (median 5 days, interquartile range 4-8 days, p < 0.0001), had a higher rate of ITU admission (24% versus 3% respectively, p < 0.0001) and a longer duration in high dependency unit (median 3 days (interquartile range 2-7 days) versus median 2 days, interquartile range 1-3 days, p < 0.0001). Univariate analyses identified differences between the patients that experienced a post-operative cardiac complication and those that did not, specifically age group (p = 0.06), history of cerebral vascular accident (CVA) (p = 0.05) (Table 1).

The distribution of the major post-operative cardiac complications across the Cardiac RCRI scores is given in Table 2, and did not correspond with those predicted from the original cohort. The extremely small number of events in the present study precludes any statistical testing being performed on its validity at predicting major post-operative cardiac complications.

**Table 2:** Major post-operative cardiac complications (excluding AF and SVT)

**Cardiac RCRI score:**	**Expected incidence of major complications**	**Post-op major cardiac complications (not AF)**		**Total**
		**Yes**	**No**	
**1**	0.5%	3 (1%)	483 (99%)	486 (69%)
**2**	1.3%	1 (1%)	149 (99%)	150 (21%)
**3**	3.6%	1 (2%)	47 (98%)	48 (7%)
**4**	9.1%	0 (0%)	19 (100%)	19 (3%)
**Total**		5 (1%)	698 (99%)	703

The frequency of all 34 (5%) post-operative cardiac complications within RCRI risk groups is displayed in Table 3. The RCRI score was a significant predictor of post-operative cardiac complications in a logistic regression analysis (χ^2^ =9.93, degrees of freedom =3, p = 0.02, Table 4). However, it had poor discriminative ability for predicting post-operative cardiac complications with an area under the ROC curve of 0.59 (95% CI 0.51-0.67) and limited predictive ability (R^2^ = 0.011).

**Table 3:** Frequency of post-operative cardiac complications (including AF and SVT) by Revised Cardiac Risk Index (RCRI) and modified Thoracic RCRI (ThRCRI) score

	**Post-op cardiac complications (including AF & SVT)**		**Total**
	**Yes**	**No**	
**RCRI score:**			
**1**	24 (5%)	462 (95%)	486 (69%)
**2**	4 (3%)	146 (97%)	150 (21%)
**3**	2 (4%)	46 (96%)	48 (7%)
**4**	4 (21%)	15 (79%)	19 (3%)
**ThRCRI score:**			
**0**	23 (5%)	474 (95%)	497 (71%)
**1-1.5**	6 (4%)	164 (96%)	170 (24%)
**2-2.5**	3 (12%)	21 (88%)	24 (3%)
**> 2.5**	2 (17%)	10 (83%)	12 (2%)
**Total**	34 (5%)	669 (95%)	703

**Table 4:** Logistic regression model for predicting post-operative cardiac complications (including AF and SVT) using Revised Cardiac Risk Index (RCRI) score

**Variable**	**Estimate**	**SE**	**p-value**	**OR**	**95% CI**	
**Intercept**	-2.96	0.21				
**RCRI score**						
**2**	-0.64	0.55	0.24	0.53	0.18	1.54
**3**	-0.18	0.75	0.81	0.84	0.19	3.66
**4**	1.64	0.60	0.006	5.13	1.58	16.65

The distribution of the post-operative cardiac complications across the modified ThRCRI scores is given in Table 3. This categorised ThRCRI score was not a significant predictor of post-operative cardiac complication in a logistic regression analysis for this whole population (χ^2^ =6.47, degrees of freedom =3, p = 0.09, Table 5). It also had worse discriminative ability than the RCRI score, area under the ROC curve of 0.57 (95% CI 0.49-0.66) and predictive ability, (R^2^ = 0.007). Its performance did not improve considerable within the select group of 452 patients having a pneumonectomy and lobectomy for which this score was developed, ThRCRI score was a borderline significant predictor of post-operative cardiac complications (χ^2^=8.00, degrees of freedom=3, p = 0.05). However it still had poor discriminative ability (area under the ROC curve 0.61; 95% CI 0.52-0.70) and predictive ability (R^2^ = 0.016).

**Table 5:** Logistic regression model for predicting post-operative cardiac complications (including AF and SVT) using modified Thoracic Revised Cardiac Risk Index (ThRCRI) score

**Variable**	**Estimate**	**SE**	**p-value**	**OR**	**95% CI**	
**Intercept**	-3.03	0.21				
**ThRCRI score**						
**1-1.5**	-0.28	0.47	0.55	0.75	0.30	1.88
**2-2.5**	1.08	0.65	0.10	2.94	0.82	10.59
**>2.5**	1.42	0.80	0.08	4.12	0.85	19.91

The performance of both the RCRI and ThRCRI scores were worse for predicting all post-operative cardiac complications or death (data not shown).

## Discussion

Major post-operative cardiac complications are rare, but serious sequelae of non-cardiac surgeries. A number of risk calculators have been developed for predicting post-operative cardiac outcome [1,2,5,6,8].

Though useful in providing risk stratification for this patient group the indexes devised by Goldman *et al.*[6] are complex and based on small patient cohorts.

For this reason Lee *et al.*[1] developed a simpler risk scoring system. The RCRI, used for predicting cardiac outcomes after non-cardiac surgery, was based on a large prospective data set (4315 patients). This identified six independent predictors (high-risk type of surgery, IHD, CCF, CVA, insulin therapy, and creatinine >2 mg/dL) for developing post operative cardiac complications [1]. Of note, the original data set contained only 343 patients who underwent thoracic surgery and provided no breakdown of what specific thoracic procedures were actually carried out.

Despite this the RCRI is incorporated into the BTS and European guidelines for patient selection for curative lung cancer surgery, in parallel with ACC/AHA guidelines for cardiovascular evaluation of patients receiving non-cardiac surgery [3,4,9]. This includes patients undergoing lung resection surgery.

In our cohort of patients, the incidence rate of major post-operative cardiac complications was lower than reported by Lee *et al.* (0.7% vs 2%) [1]. Therefore no useful analysis could be performed. This may have been expected because in the Lee *et al.* paper thoracic surgery with a relative risk of 1.1 for post-operative cardiac complications was combined with abdominal aortic aneurysm, other vascular and other high risk surgeries with relative risks of 3.6, 3.9, and 2.1 respectively. It seems likely that the risk of major complications is lower than predicted by RCRI score. The new UK and European guidelines for the radical management of patients with lung cancer recommend cardiologist review when three or more risk factors are present.

In the highest risk groups (RCRI >3), 27.9% developed a post-operative pulmonary complication (19/68 patients), of which one died (there was one further death in the study population). Six high risk patients developed cardiac complications post-surgery (one AF and MI, four with AF and one had a MI alone). The rate of cardiac complications post-surgery in the highest risk groups was 8.8%.

In our cohort this would equate to 10% of lung resection surgery patients needing to see a cardiologist prior to surgery even if they were asymptomatic. This increase in referral pattern would lead to an unnecessary burden on the system and potentially could lead to unacceptable delays in performing curative lung cancer surgery. This fact reinforces the need for a robust and effective risk score that can easily be applied by a non-cardiologist.

The validity of including new onset AF with major cardiac complication is based on the significant ensuing morbidity. Up to 20% of patients develop atrial fibrillation following thoracic surgery and it is associated with an increased rate of post-operative complications [4]. Increasing age, increasing extent of operation, male sex, Caucasian race, and advanced clinical disease (Stage II or greater) are significant independent risk factors for developing AF following thoracic surgery [4]. Developing an arrhythmia post thoracic surgery is associated with an increase in length of hospital stay [10,11] and reduced long term survival [8]. In our study, those who developed AF had longer hospital and HDU length of stay. Sixty percent of patients who developed AF also developed a post-operative pulmonary complication. Predicting those at high risk of developing AF would allow targeting of preventative strategies. Recent guidelines on the management of atrial fibrillation in cardiac and non-cardiac patients do not include risk stratification [12]. Although a risk score for AF following cardiac surgery has been described [13]. There is no validated predictive tool for AF in non-cardiac surgical patients nor has any paper described RCRI as predictor of AF. However the risk factors for AF in part overlap with those of RCRI thus one might infer that the RCRI might be useful in predicting which patients develop AF [14].

The RCRI score was a significant predictor of post-operative cardiac complications but failed to show any discriminative ability with an area under the ROC curve for the RCRI score of 0.59 (95% CI 0.51-0.67). This inability of RCRI to accurately predict cardiac events [15,16] and/or mortality [17,18] has also been noted after vascular surgery. Thus, Brunelli *et al.* recently recalibrated the RCRI for patients undergoing lung resection and subsequently devised the ThRCRI. The aim was to provide a more discriminatory cardiac risk stratification [2]. In their study, the authors included 1426 patients who underwent lobectomy and 270 pneumonectomy patients and tested the six independent predictors devised by Lee *et al.*[1].

In the ThRCRI score, only four of the original six variables were found to be significant independent predictors of post-operative cardiac events. The ThRCRI score achieved higher discriminative factor than the original RCRI (C index; ThRCRI 0.72 vs RCRI 0.62). The authors and a further independent group validated ThRCRI on a test cohort and concluded that it was a good discriminator in stratifying for risk of cardiac complications after lung resection surgery (ROC area of 0.75 and 0.736 respectively) [19,20].

In their study, despite lower incidence of co morbidity (CVA: 4% vs 9%; renal disease: 3.3% vs 9%), the incidence of major cardiac complications (not SVT) was 3.3% which is much higher than we report (<1%). These differences may in be part be due to the inclusion of only lobectomy or pneumonectomy patients when compared to our sample which included lesser resections [2]. Our study failed to show that the ThRCRI score could accurately predict all postoperative cardiac complications including AF.

Due to the lack of detailed information regarding the intercept and regression coefficients of the original model we were unable to undertake a thorough external validation of these indices and update the existing risk indices.

We acknowledge our reported complication rate is lower than some other reported series [1,2,20]. This difference may be in part be explained because we included only complications as defined in the original paper [1] and did not include or search for isolated enzyme rises.

## Conclusions

Although major postoperative cardiac complications lead to increased morbidity and mortality, they remain rare. In this observational cohort study the RCRI and ThRCRI scores failed to accurately predict major cardiac complications and atrial fibrillation following elective lung resection surgery. It would be important to validate these findings in a multicentre study. The larger numbers would also allow for a multivariate analysis of factors associated with development of these complications, which was not possible in this study.

## Abbreviations

ACC/AHA: American College of Cardiology/American Heart Association; AF: Atrial fibrillation; ASA: American Society of Anaesthesiologists; BTS: British Thoracic Society; CCF: Congestive heart failure; COPD: Chronic obstructive pulmonary disease; CVA: Cerebrovascular disease; FEV1: Forced expiratory volume in one second; ppoFEV1: Postoperative predicted FEV_1_; HDU: High dependency unit; IHD: Ischaemic heart disease; ITU: Intensive care unit; MI: Myocardial infarction; RCRI: Revised cardiac risk index; ROC: Receiver operator characteristic; ThRCRI: Thoracic revised cardiac risk index; VF: Ventricular fibrillation.

## Competing interests

The authors declare that they have no competing interests.

## Authors’ contributions

AK was involved in study design, data collection and drafted first manuscript. AM carried out the statistical analysis. RW was involved in draft revisions and manuscript preparation for publication. EB and MK participated in both study design and data collection. RS participated in data analysis. PR involved in drafting first manuscript. BN conceived the study, before co-writing initial draft and approving all subsequent versions. All authors read and approved the final manuscript.
