# Erratum to: Does the revised cardiac risk index predict cardiac complications following elective lung resection?

**DOI:** 10.1186/s13019-014-0182-9

**Published:** 2014-11-26

**Authors:** Mahmoud Abdelaziz, Andrea Marshall, Amy Kerr, Khalid Hussain, Robin Wotton, Ehab Bishay, Maninder Kalkat, Pala Rajesh, Richard Steyn, Babu Naidu

**Affiliations:** Department of Thoracic Surgery, Heart of England NHS Foundation Trust, Bordesley Green East, Birmingham, B9 5SS UK; Warwick Medical School Clinical Trials Unit, Warwick Medical School, University of Warwick, Coventry, CV4 7AL UK; University of Birmingham, Office 8 First Floor Research Laboratories, Mindelsohn Way, Edgbaston, Birmingham B15 2WB UK

## Abstract

No abstract

After publication of this work [1], it was noted that Mahmoud Abdelaziz and Khalid Hussain had been inadvertently omitted from the author list. The list of authors has now been updated and the order amended. The Authors’ Contributions and Competing Interests section have been modified accordingly.
